# Polyamide 6-Aluminum Assembly Enhanced by Laser Microstructuring

**DOI:** 10.3390/polym14020288

**Published:** 2022-01-11

**Authors:** Karol Bula, Bartosz Korzeniewski

**Affiliations:** Institute of Materials Technology, Faculty of Mechanical Engineering, Poznan University of Technology, PL-60965 Poznan, Poland; bartosz.korzeniewski@doctorate.put.poznan.pl

**Keywords:** polyamide 6, aluminum, hybrid joints, overmolding, laser structuring, mechanical properties

## Abstract

The presented work’s aim is the application of low-power laser treatment for the enhancement of interfacial micromechanical adhesion between polyamide 6 (filled with glass fiber) and aluminum. A fiber laser beam was used to prepare micro-patterns on aluminum sheets. The micro-structuring was conducted in the regime of 50, 100, 200 and 300 mm/s laser beam speeds, for both sides. The joining process was realized in an injection molding process. Metallic inserts were surface engraved and overmolded in one-side and two-side configurations. A lap shear test was used to examine the strength of the joints. Engraved metallic surfaces and adequate imprints on polyamide side were checked by optical microscope with motorized stages, and roughness parameters were also determined. Microscopic observations made it possible to describe the grooves’ shape and to conclude that a huge recast melt was formed when the lowest laser beam speed was applied; thus, the roughness parameter Ra reached the highest value of 16.8 μm (compared to 3.5 μm obtained for the fastest laser speed). The maximum shear force was detected for a sample prepared with the lowest scanning speed (one-sides joints), and it was 883 N, while for two-sided joints, the ultimate force was 1410 N (for a scanning speed of 200 mm/s).

## 1. Introduction

Recently, in the emerging field of lightweight construction, a lot of new attempts have been made in which stiff metal parts are joined with engineering thermoplastic materials, without the use of a third, adhesive-like component. Various methods have been utilized to gain mechanical interlocking between polymeric parts and metal surface. These methods are majorly connected with mechanical coupling where molten polymer forms rivet joints in the through-holes of the metallic part [1,2,3]. Among the novel versions of rivet-like connections are those that are based on friction spot welding (FSW) [4,5,6] and the friction filling staking joining technique (FFSJ) [7]. Mechanical fastening and the production of rivet-like connections have potential disadvantages. For example, using single contact point fastening would cause stress concentrations near the hole/rivet and might reduce the flexibility of the work piece design. There are few novel methods used for forming polymer–metal hybrids that differ from the classic ones, with the existing ones including: In-Mold-Assembly or Post-Mold-Assembly. Moreover, most of them are focused on additional operations with a third machining stage, such as, for example, clinching [8,9], ultrasonic and hot air stacking [10] and the fused deposition modeling (FDM) method [11]. 

However, overmolding through injection molding is still the most versatile method for the multiplication of such bimaterial elements, and it is easily applicable for household goods, as well as automotive and electronic applications. The most significant advantage of the In-Mold-Assembly method is that the joint between the polymer and the metal is established in the same production step as that during which the polymer part is formed [12,13]. For this conventional method, one condition should be fulfilled to strengthen the joint of the biomaterial element. The condition is related to ability of molten polymer to infiltrate metal surfaces that were previously microstructured or riveted [14,15]. This means that the enhancement of metal–polymer joint strength is proportional to the real area of contact on the interphase. In scientific approaches, there are many other factors that determine the success of a bimaterial joint, including chemical affinity, processing parameters (such as mold heating during the filling stage), injection speed, holding time and pressure, etc. [16,17,18,19].

Discussing only first hypothesis, which deals with metal structurization, researchers attempted to evaluate not only the roughness of the etched, blasted or patterned metal surface, as was the case for Lucchetta et al. [20], but they also considered possible differences between the longitudinal and transversal orientations of pattern traces, such as the study of polymer flow direction conducted by Rodriguez-Vidal et al. [21]. Some other authors presented various individual parameters that could be linked directly to the effectiveness of the processed overmolding part. Schricker et al. introduced a new parameter, known as the penetration parameter, based on an investigation of the interaction between the geometrical parameters and shear strength of laser-structured Aluminum 6082 and Polyamide 66 [22]. All of the examined parameters that were found to characterize the metal structured surface (the width and depth of grooves, and the structural density) can be reduced to the significant influence of the depth. Even more importantly, the theoretical loadable area was not found to be an important factor with respect to shear strength. Zhao et al. analyzed the mechanical interlocking in a bimaterial joint made of aluminum sheets that were micro structurized by pulse laser treatment and overmolded by polybutylene terephthalate (PBT) [23]. The authors investigated the correlation between the applied processing parameters and the real contact between the metal and the polymer, which was ascribed to the infiltration depth. The infiltration depth was defined as the distance between the metal surface and the polymer head, and represented the depth to which the molten polymer flowed in the surface dimples. This parameter was simply revealed by dissolving the aluminum part. The results showed that the infiltration depth (measured in μm) was mostly affected by the packing pressure and polymer plasticizing temperature. Moreover, the fitted correlation coefficient between the infiltration depth and the joining strength was high, at 0.785 μm. All other tests proved that the fracture was due to the breakage between the infiltrated polymer and the polymer’s main part. The final conclusion given by the authors was that the failure mode was more cohesive (the most expected behavior) when the infiltration was deeper. Consistent findings were also presented in two other articles [24,25].

Rodriguez-Vidal et al. produced different micro-pattern geometries on HC420 steel sheet before it was subjected to overmolding with PA6-GF30 (glass fiber-reinforced polyamide 6) [26]. They used a fiber laser operated in cw (continuous wave) mode (a laser beam speed of 360 mm/s). They analyzed the groove geometry and recast the material height in relation to the number of laser beam repetitions on the same groove. Finally, the development of the failure force was presented against the aspect ratio (AR) factor of the micro-patterns, which was defined as the ratio of their depth (d) to width (w). They concluded that higher aspect ratios of the patterns caused greater joint strength, until the AR reached the limit point. In their experiment, the AR limit value was around 3. Above that point, the failure force remained constant. 

In this study, we proposed another issue concerning the efficiency of laser micro pattering as a method to be used for high speed metal structuring before overmolding with plastic materials. The intention of this work was to resolve the most interesting topic, which is associated with verifying the relation between the chosen laser working parameters and the effect on surface topography, and finally, on the joining strength properties. The laser-engraved surface was aligned in the transverse direction in relation to the polymer flow direction during the overmolding process. The efficacy of laser treatment was confirmed by means of lap shear tests together with microscopic observations of the engraved surface. The observations provided additional information that may be attractive in terms of the utilization of fast, contactless methods of treatment in the preparation of metal surfaces dedicated to overmolding with engineering plastics.

## 2. Materials and Methods

### 2.1. Materials

As a polymer material, Tarnamid^®^ T-27 GF30 (manufactured by Grupa Azoty, Tarnów, Poland) was chosen. It is a polyamide 6 thermoplastic polymer, reinforced with chopped fiberglass that contributes 30 percent of its mass, along with the addition of thermal stabilizer. The material has a mass flow rate (MFR) of 34 cm^3^/10 min at 260–280 °C. The pellets were dried before use at 80 °C over 24 h.

Metal inserts were made of the precipitation-hardened aluminum alloy 6061, in T6 temper condition, due to the relatively high strength it exhibits. Alloy 6061 is commonly used in the automotive and aviation industries, which made it a good candidate for the experiment.

### 2.2. Preparation of Metal Inserts

**Figure 1:**
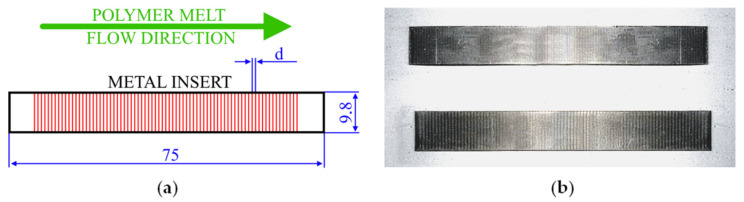
Metal inserts: (**a**) shape, dimensions, and alignment of laser-engraving in relation to polymer melt flow direction; (**b**) example inserts with different distances between grooves.

Metal sheet made of 6061 aluminum alloy was cut into inserts with 75 × 9.8 × 1 mm dimensions after the entire sheet was laser engraved (Figure 1). For the laser-engraving procedure, the Wisely MY-M20F-III (manufactured by Wisely Laser Machinery Limited, Shenzhen, China) device was used. It is a diode laser working in pulsed mode. Different laser-scanning speeds and distances between grooves were chosen. The engraving parameters are shown in Table 1, while all sample types are listed in Table 2.

The laser-engraved surface consisted of grooves with expelled material (recast), which mostly formed on groove shoulders (edges). To remove weakly attached recast material, the ultrasonic cleaning method was used. Cleaning was done in Ulsonix Proclean 0.7 M (manufactured by Expondo GmbH, Berlin, Geramany) device. The metal inserts were bathed in acetone heated to 70 °C, for 30 min.

### 2.3. Surface Roughness Profiles

The engraved inserts had their surfaces scanned with an SRT-220 surface roughness tester in order to measure the shape and parameters of grooves and recast material, and to further correlate the results with later microscopic observations. Finally, the same measurements were made on actual joints, after destructive tests had been conducted, to check how bimaterial coupling cooperated during shear loading. 

### 2.4. Microscopic Observations

Metal insert surfaces and cross-sections were observed through the Nikon Eclipse MA200 (Nikon Instruments, Tokyo, Japan) reflecting microscope, used alongside a digital camera and motorized stages. Combined with Nikon Elements Basic Research software, the usage of Real Time EDF (Extended Depth of Focus) functionality was made possible, allowing the production of images with very high depth through combining images captured in different focal planes. In addition, this mode allowed to generate 3D surface models of such 2D images.

### 2.5. Overmolding Procedure

Polyamide 6-aluminum hybrid joints were made by means of an overmolding process conducted using the ENGEL ES 80/25 HLS (Engel Austria GmbH, Schwertberg, Austria) type injection molding machine, in which the mold had rectangular shaped cavities with thicknesses of 2 mm and 3 mm. The process parameters are shown in Table 3.

**Figure 2:**
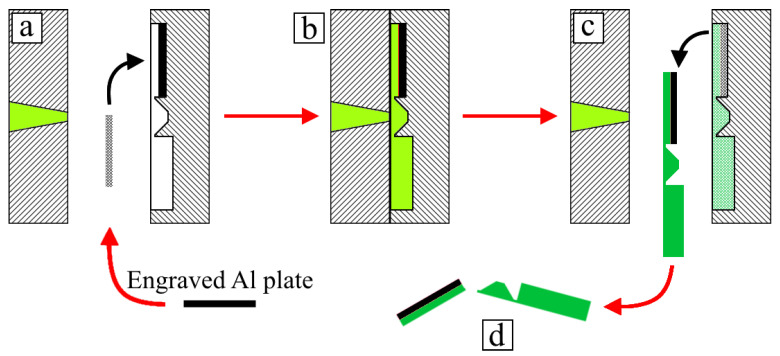
Overmolding process of one-sided joints: (**a**) placement of metal insert into mold cavity; (**b**) polymer melt’s injection; (**c**) molding’s removal; (**d**) separation of joint from sprue.

**Figure 3:**
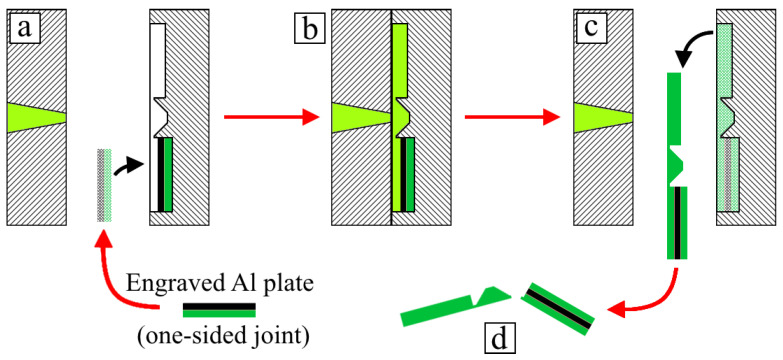
Overmolding process of two-sided joints: (**a**) placement of one-sided joint into mold cavity, which is deeper than the previously used cavity; (**b**) polymer melt’s injection; (**c**) molding’s removal; (**d**) separation of joint from sprue.

**Figure 4:**
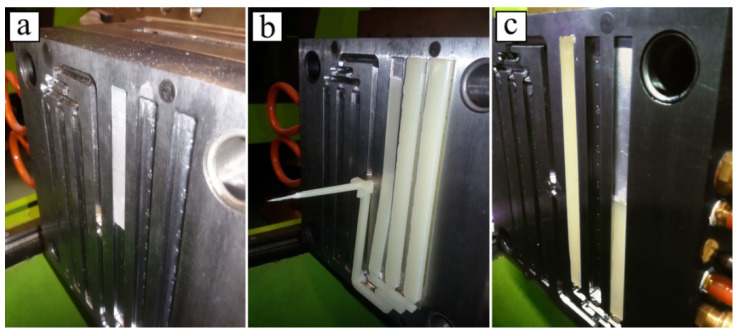
Example stages of overmolding process: (**a**) metal insert is placed into mold cavity; (**b**) molding is ready for removal from the mold; (**c**) one-sided joint is inserted into a deeper mold cavity.

One-sided joints were made by placing a metal insert into the mold cavity and performing the injection molding procedure, followed by the removal of the overmolded inserts from the mold (Figure 2). To produce two-sided joints, previously made one-sided joints were used as inserts, but were placed in mold cavities in such a way that the metal part’s surface was exposed for the next polymer injection (Figure 3). For process visualization, selected stages were captured in images, and these are displayed in Figure 4.

### 2.6. Mechanical Testing

**Figure A1:**
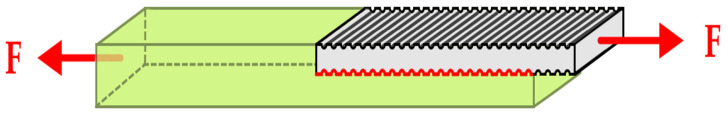
Simplified sketch of bi-material sample visualization during the lap shear tensile test of the one-sided joint (green color represents polyamide 6 GF 15, while gray indicates the engraved aluminum plate).

A sketch of the sample intended for use in tensile shear testing is presented in Figure A1. Bicomponent lap joints were shear tested using a Zwick/Roell Z010 (Zwick/Roell, Ulm, Germany) universal tensile testing machine. A starting force of 5 N was applied with test speed of 2 mm/min. Tests were carried out with mechanical wedge grips.

### 2.7. Polyamide Coating with PVD Method 

After shear testing, the polymer parts of bicomponent joints needed to have their surfaces modified with microscopic observations in mind; this due to the high visual light transparency of the fiberglass-reinforced polyamide 6. For this reason, the PVD (Physical Vapor Deposition) method was employed using magnetron sputtering. A thin metallic layer was deposited by utilizing a WMK-100 circular magnetron gun (Division of Vacuum and Plasma Technologies, Wroclaw University of Technology, Poland). The process used copper as a target and argon as a working gas. The parameters of coating process were as follows: pressure inside chamber: 9 × 10^−3^ hPa; electrode current: 3 A; deposition power density: 10 W/cm^2^; deposition time: 30 s. The thickness of the copper coating was low enough to not affect the topography of the coated surfaces, allowing accurate microscopic observations.

## 3. Results and Discussion

**Figure 5:**
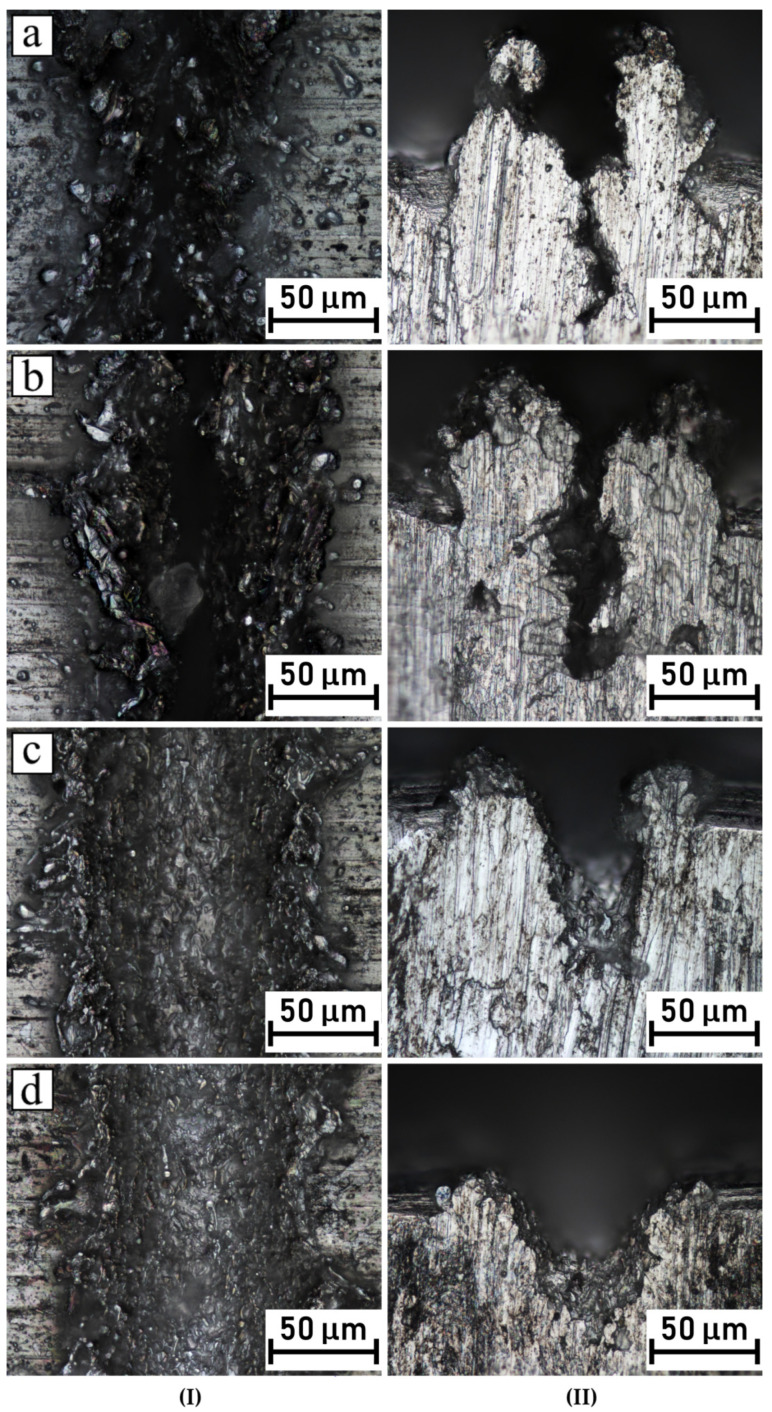
Top-down (**I**) and cross-section (**II**) microscopic observations of grooves in Al inserts engraved with varied laser-scanning speeds: (**a**) 50 mm/s, (**b**) 100 mm/s, (**c**) 200 mm/s, (**d**) 300 mm/s.

Different micro-pattern geometries were produced by the changing laser beam scanning speed and the distance between each laser beam trace. An important remark should be addressed here. The grooves’ geometry, as well as their width, depth and recast material height, were expected to be in close relation with the contact time of the laser beam and the local specific area that was under thermal excitation, caused by energy concentration. Thus, was expected that patterns scanned at low laser beam speeds would reveal quite rough surfaces with relatively high volumes of recast metal melts. On the other hand, patterns etched at high laser speeds typically possess smooth surfaces, where melted metal does not form rough recast residues. Therefore, microscopic observations of the cross sections of the micro-patterns as well as of the roughness profiles were carefully performed. Figure 5 shows the metallographic photos (plain and cross section view) of single grooves processed at various laser-scanning speeds. The observed pictures make possible some conclusions about the groove shape formation trend. It can be clearly seen that, at low laser speeds, a narrow groove shape was formed with huge participation of the generated recast melt, not only on the top of profile, but also at the shoulders of the formed grooves, shown on Figure 5a,b. Moreover, it should be noted that the root canal in the center of the groove was very narrow. It is likely that the intensity of the heat beam allowed for the melting of a huge amount of aluminum in that precise location and, after that, the melted aluminum was frozen by the surrounding cold bulk material. The described effect led to the restriction of the further penetration of very narrow grooves by the polymer melt. The groove shaped under moderate laser speed (v = 200 mm/s) represents an intermediate nature of the cross section, with an open U-shaped bottom and a recast melt that was less formed, as shown on Figure 5c. A contrasting situation was observed when the scanning speed of the laser beam was fast (v = 300 mm/s). As a consequence, grooves were characterized by very wide bottom and less or marginal presence of shoulders formed by the recasting of the melt. This is especially well revealed on Figure 5d. Rodriguez-Vidal et al. documented and described a very similar situation after the laser treatment of HC420 steel [26]. They used the same laser speed (v = 360 mm/s), but they changed the number of trajectory repetitions (from 2 times up to 18 times). With more repetitions, the recast metal volume increased and the entrance to groove became very narrow. 

**Figure 6:**
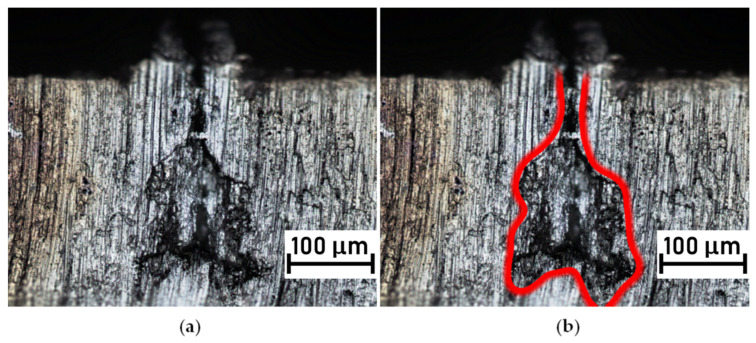
Microscopic observations of metal inserts engraved with 50 mm/s scanning speed: (**a**) groove cross-section with visible necking, (**b**) actual groove with red outline.

As mentioned earlier, when the laser beam was tracked on the aluminum sheet with the lowest speed (v = 50 mm/s), a huge recast melt was produced. As a result, the nature of the formed grooves was rather narrow (Figure 6a). Some of the observed grooves fabricated at the same laser speed revealed specific shapes, which are visible in the cross section shown in Figure 6a. The groove was built with a narrow neck at the top and some spacious volume below the neck. To highlight the individual character of this groove, its shape was outlined by a red line, which is shown on Figure 6b. This method made it possible to identify the groove contours as an irregular geometry. Moreover, it was found that the irregularly shaped bottom part of the groove (covered by the outer recast) can be filled by the polymer melt only if the polymer is able to infuse throughout the very narrow neck. Rodriguez-Vidal et al. acknowledged a similar formation of groove shapes in laser-microstructured steel, but only when the number of laser beam tracks reached 18 repetition [26]. Even then, the shape of the bottom part of the groove was more regular.

**Figure 7:**
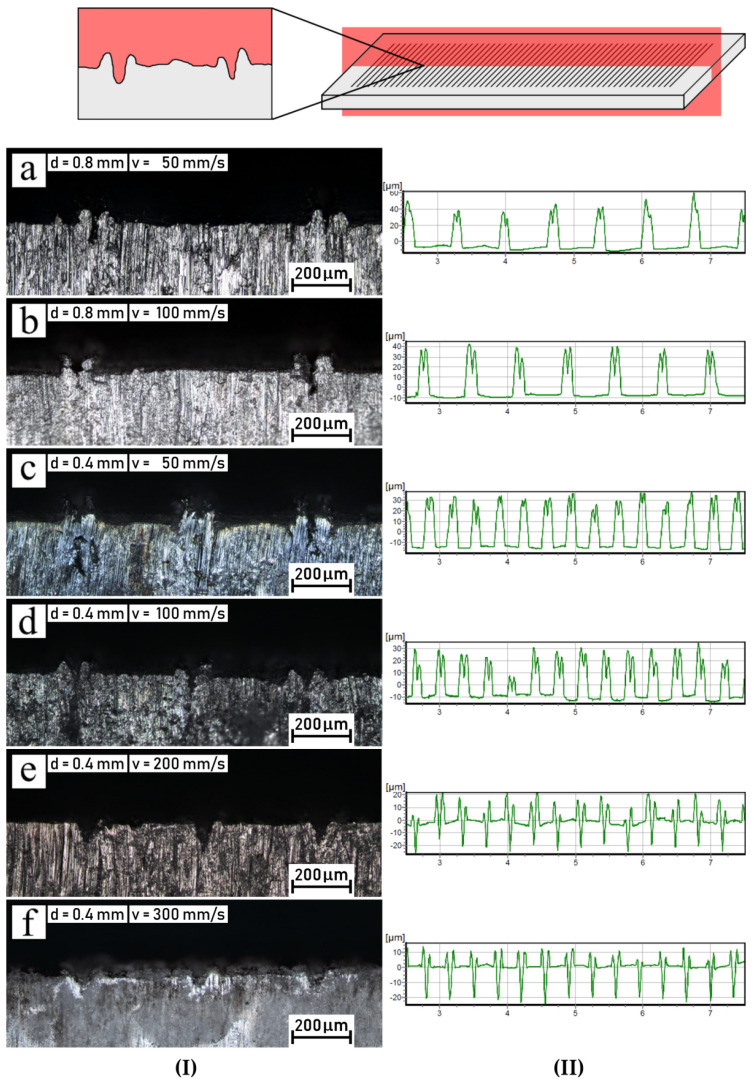
Metal inserts engraved with various parameters (space between grooves *d* and scanning speed *v*): (**a**) d = 0.8 mm, v = 50 mm/s; (**b**) d = 0.8 mm, v = 100 mm/s; (**c**) d = 0.4 mm, v = 50 mm/s; (**d**) d = 0.4 mm, v = 100 mm/s; (**e**) d = 0.4 mm, v = 200 mm/s; (**f**) d = 0.4 mm, v = 300 mm/s. (**I**) Cross−sections; (**II**) surface roughness profiles.

In Figure 7, pictures of microstructural cross sections of aluminum, with distances of 800 μm (Figure 7a,b, column I) and 400 μm (Figure 7c–f, column I) between grooves, together with corresponding roughness profiles (Figure 7, column II), are shown. As can be seen in the pictures included in Figure 7, the geometry of the grooves engraved with 800 μm distance represents the same shape contour as those that are adequate for 400 μm distance. Narrow grooves are still present in the pictures of aluminum intended for laser treatment at the lowest beam speed. Thus, it can be assumed that laser treatment did not affect the interactions between the ablated traces. Roesner et al. revealed that the application of a μs-pulse laser system with 40 W power and a laser frequency of 22.5 kHz (feed rate 450 mm/s) led to noticeable interactions between adjacent structures and influenced the microstructural geometry [27]. They observed that disadvantageous behavior took place for interline distances of 200 µm. 

In column b of Figure 7, corresponding roughness profiles are presented. The shape of the groove profiles shown in Figure 7a–d (column II) indicates that the application of the needle of the contact tester did not track into the internal part (below the neck) of the groove. Therefore, the needle path probably only recorded the top of the neck zone, without penetration inside it. As a result, the depths of the grooves engraved with laser speeds of 50 mm/s and 100 mm/s could not be measured. However, it can be deduced that the height of the formed recast decreased from 40 µm up to 20 µm for laser speeds of 50 mm/s and 100 mm/s, respectively. A contrasting shape was shown by the traces of roughness for the grooves that were engraved with higher laser-scanning speeds, as shown in Figure 7e,f (column II). For these samples, the depth of the grooves could be recognized and measured because the recast shoulders were minor and the needle was able to track the bottom of the grooves. In both cases, the grooves’ depth was around 20 μm. Moreover, the samples treated with laser speeds of 300 mm/s exhibited less remaining recast, with around 10 μm height. Meanwhile, the top points recorded on the surface scanned with a laser speed of 200 mm/s were twice as high as in the previous one.

The roughness characteristics obtained for all engraved aluminum sheets are presented in Table 4, along with groove dimension measurements. The most recognized parameters concerning roughness Ra (μm) and Rz (μm) decreased gradually with the increasing of the laser-scanning speed. This phenomenon was directly linked with the time of metal ablation, which was comparable to the laser beam speed. Less energy transferred into the metal corresponded to lower roughness parameters of the micro-structurized aluminum surface. Very similar relationships between surface roughness and laser-scanning parameters for the Al alloy 5182 were investigated by Han et al. [28]. They used a pulse-mode working laser, at a frequency of 100 kHz, a nominal power 100 W and a scan speed of 700 mm/min. The number of laser-scanning repetitions was set at 1, 5, 10, 20 and 40 times. An adequate surface roughness, comparable to that generated in our experiment by the pulsed laser system at 50 mm/s speed, was obtained for 10 repetitions of application of the laser beam, with the surface roughness value being 13.76 μm (Ra).

**Figure 8:**
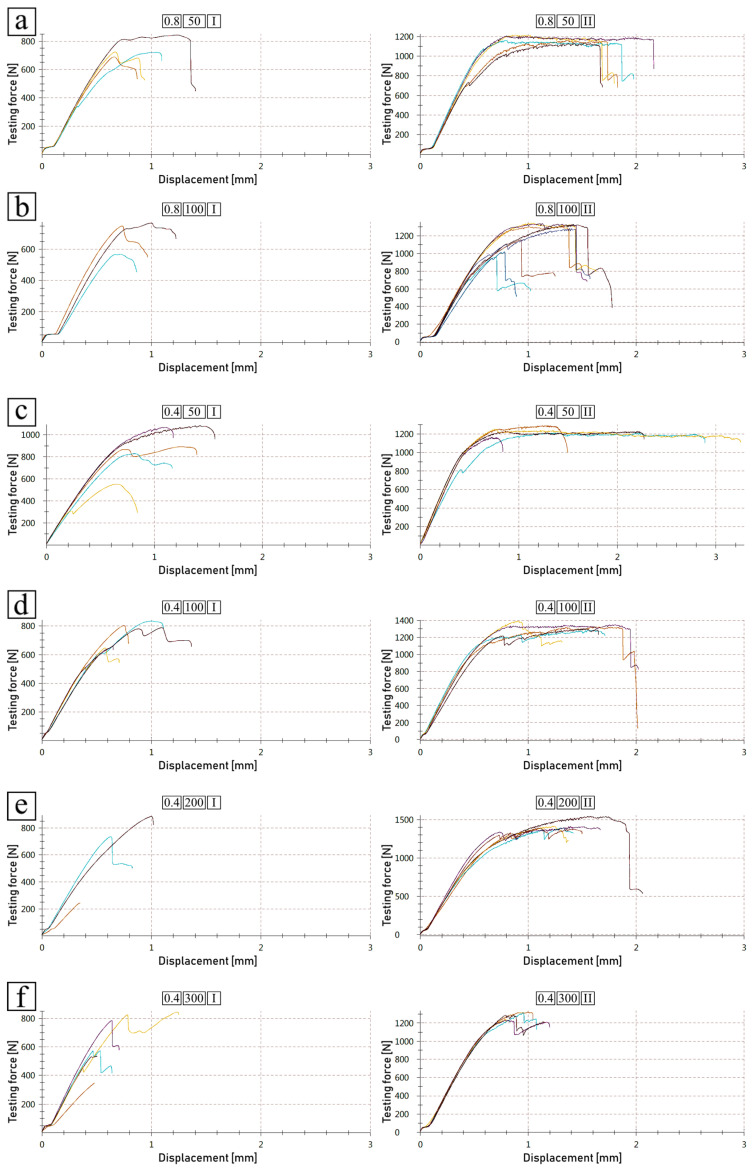
Shear-tested joints of various parameters (space between grooves *d* and scanning speed *v*): (**a**) d = 0.8 mm, v = 50 mm/s; (**b**) d = 0.8 mm, v = 100 mm/s; (**c**) d = 0.4 mm, v = 50 mm/s; (**d**) d = 0.4 mm, v = 100 mm/s; (**e**) d = 0.4 mm, v = 200 mm/s; (**f**) d = 0.4 mm, v = 300 mm/s. Each graph is labeled with squares, in left-to-right order: distance between grooves, scanning speed, joint type (one-sided (**I**), two-sided (**II**)).

**Figure 9:**
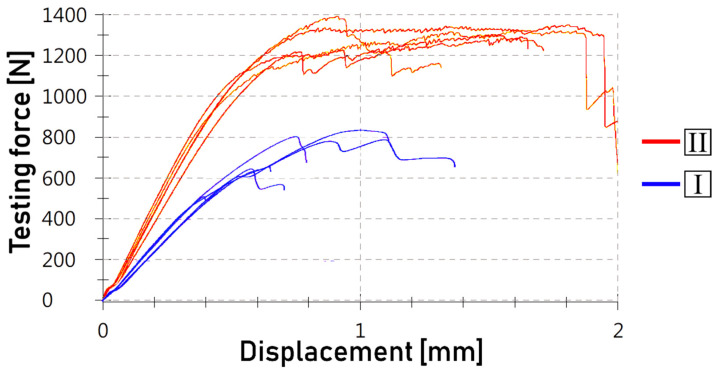
Comparison of shear test curve shapes of one-sided (**I**) and two-sided (**II**) joints. Take note of the serrated character of the two-sided joints. The example is based on parameters of v = 100 mm/s and d = 0.4 mm.

The shear test results are aggregated in Table 5, while graphs are included in Figure 8 and Figure 9. Attention was paid to the ways in which the distances between grooves, scanning speed and type of joint (one- or two-sided) affected the joint’s strength.

**Table 4:** Sample variants defined by differences in working laser parameters.

Distance between Grooves (mm)	Scanning Speed (mm/s)	Ra (μm)	Rz (μm)	Recast Height (μm)	Recast Width (μm)	Groove Depth ^1^ (μm)	Groove width ^1^ (μm)
0.4	50	16.829	53.969	50	46.7	98.7	21.9
	100	12.106	46.180	100	39.7	111.5	26.3
	200	4.955	47.747	200	15.3	108.9	51.1
	300	3.589	37.700	300	11.7	125.9	35.0
0,8	50	13.213	66.874	50	42.1	114.4	33.5
	100	11.865	50.628	100	53.0	115.7	4.8

^1^ Laser-engraved grooves or grooves in recast material that obstructs them; refer to Figure 10, Figure 11, Figure 12 and Figure 13.

A reduction in the distance between grooves from 0.8 to 0.4 mm meant that there were two times as many grooves and recast shoulders, where both contribute to the joint strength. This implied an increase in strength of around double. This, however, did not prove to be true, as the strength increase did not exceed 40%; instead it was usually around 20% or even 10%, depending on other parameters. However, the values of the strength at break were noticeably higher, with increases ranging from around 25% to 60%.

When analyzing the effect of the laser-scanning speed on the groove’s morphology and, subsequently, on the resulting shear strength, it became apparent that the laser speed parameter played a significant role in the joint’s performance. Firstly, recast material did in fact contribute to the joint’s strength. The higher the recast, the higher the shear strength. However, the results show that the grooves with exposed bottoms (no neck), in spite of the small recast amounts, provided much higher shear strength. It was noticed that grooves etched with the highest scanning speed (300 mm/s) provided slightly smaller strength increases when compared to another group of fast-etched samples (200 mm/s). This was likely due to the much lower depth of the groove. All of this means that it was the grooves’ depths and inner surfaces that played the most crucial role in joint strength, while the recast material may have partially assisted in the improvement of the strength.

Finally, one-sided and two-sided joints were compared. While two-sided joints did essentially have doubled surface areas, in the lap shear test, it did not appear that the joint strength simply doubled. The resulting strength seemed to be highly dependent on other, previously discussed parameters. Combining low scanning speed (which produced high recast with narrow necks) with two-sided joints yielded increases in strength of only around 60% to 80% with regard to one-sided joints, which was counter-intuitive—based on the surface area, there should have been a 100% increase. However, the two-sided joints benefitted significantly from higher scanning speeds (which produced grooves with shallow yet exposed bottoms), providing increases in shear strength from around 105% to 130%. This means that, with proper parameters, the strength of a two-sided joint can noticeably exceed that which would be achieved with a one-sided joint when both have the same surface area.

In addition to the observations described above, shear test graphs revealed another interesting feature of the two-sided joints. When analyzing graphs shown on Figure 8, it is apparent that not only the force- and stress-related aspects were affected, but also the displacement. With regard to one-sided joints, there was an increase in displacement of at least half before breakage occurred in all tested two-sided joints, regardless of other parameters. The shapes of the curves changed significantly as well; it can be noticed that the two-sided joint curves were flatter after reaching the maximum force value, meaning that the load needed to be maintained for further displacement and for breaking to eventually occur, while in the one-sided joints, there was a steep drop in the load required to progress with the test. Both the displacement range and the curve overall shapes may be explained by analyzing Figure 9, which compares the one-sided joints with the two-sided joints in one graph. It can eb seen that the two-sided joints had curves of a serrated-like shape. This possibly shows that the polymer was separated from the grooves in a long sequence, one by one, rather than in large areas, which was likely the case with the one-sided joints.

**Figure 10:**
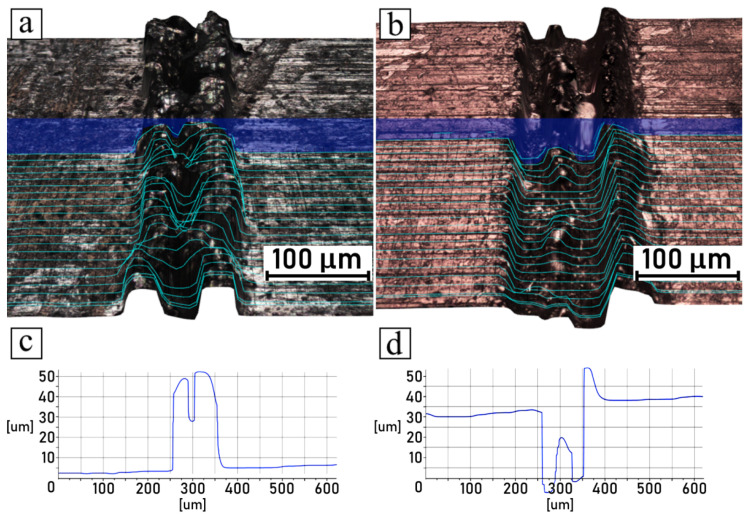
Shear-tested joint engraved with 50 mm/s scanning speed: (**a**) 3D surface model of metal part; (**b**) 3D surface model of polymer part; (**c**) groove cross-sectional profile of metal part; (**d**) groove cross-sectional profile of polymer part.

**Figure 11:**
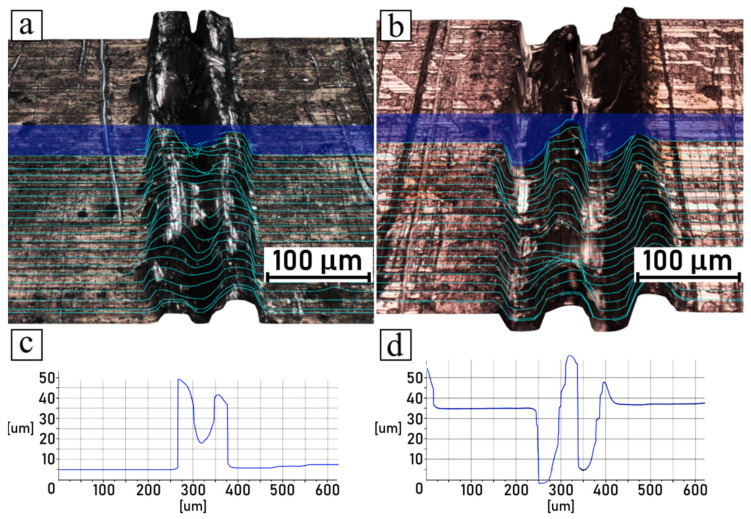
Shear-tested joint engraved with 100 mm/s scanning speed: (**a**) 3D surface model of metal part; (**b**) 3D surface model of polymer part; (**c**) groove cross-sectional profile of metal part; (**d**) groove cross-sectional profile of polymer part.

**Figure 12:**
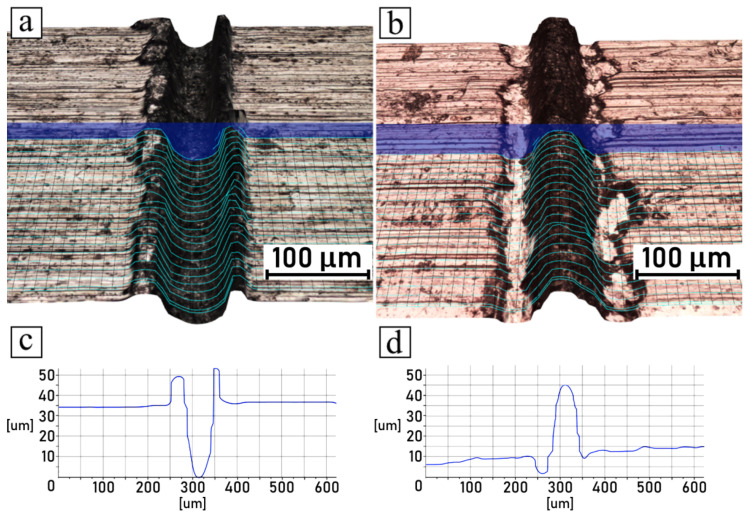
Shear-tested joint engraved with 200 mm/s scanning speed: (**a**) 3D surface model of metal part; (**b**) 3D surface model of polymer part; (**c**) groove cross-sectional profile of metal part; (**d**) groove cross-sectional profile of polymer part.

**Figure 13:**
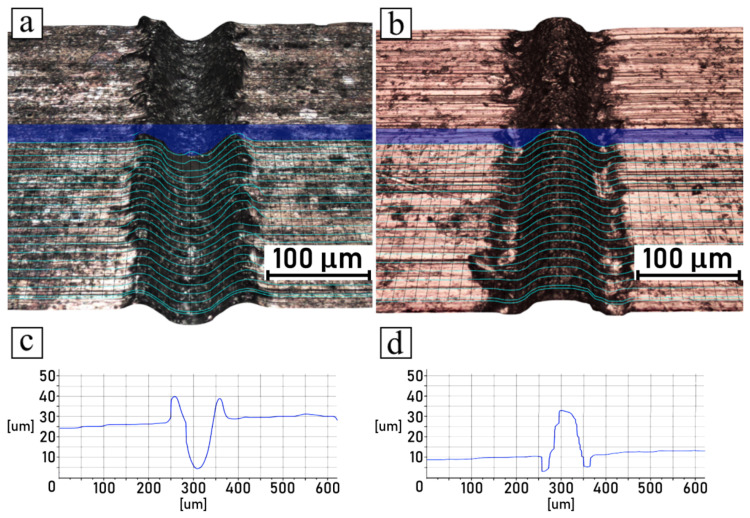
Shear-tested joint engraved with 300 mm/s scanning speed: (**a**) 3D surface model of metal part; (**b**) 3D surface model of polymer part; (**c**) groove cross-sectional profile of metal part; (**d**) groove cross-sectional profile of polymer part.

The microscopic observations of samples conducted after the shear tests are summarized in Figure 10, Figure 11, Figure 12 and Figure 13, which compare the surfaces of the metal parts and the polymer parts after their separation through shear testing. In this case, the sample surface of the polyamide 6 GF 30 was coated with a thin layer of copper by means of the magnetron sputtering deposition technique, described in Section 2.7. Images were taken from the same samples, but not from the exact corresponding areas; however, the high quality of the engraved patterns ensured good comparability. Moreover, visual observations conducted in 3D mode were supplemented with cross-sectional profiles of each detached part, for both the metal and plastic parts. The pictures shown in Figure 10a,b, Figure 11a,b, Figure 12a,b and Figure 13a,b are 3D scans of the observed surfaces. To improve the readability of the 3D scans, contour lines were generated on the surfaces, along with cross-sections in one cutting plane, as shown in Figure 10c,d, Figure 11c,d, Figure 12c,d and Figure 13c,d. Due to the high levels of irregularity in terms of the colors and shapes of both the metallic and polymeric parts, the algorithm responsible for the generation of the 3D scans was often inaccurate, which resulted in mistakes that were noticeable. Therefore, these scans should not be considered as direct representations of the measured surfaces, but rather as generalized visualizations. To limit the inaccuracies, several images were scanned and analyzed, and then the most accurate ones (judging on the basis of live microscopic observations) were selected. Consequently, the cross-section profiles were also manually filtered and the most frequently occurring ones were selected.

The analysis of discussed pictures revealed that, regarding the chosen parameters, the samples did not break cohesively, but rather adhesively. The polymeric part was pulled out of the grooves, which was evident from the polymeric part surfaces having negative shapes in relation to the metal surfaces, which remained true with the high recast samples as well. Additionally, no traces of polymer were found in the metal grooves.

## 4. Conclusions

The laser micro-structuring technique with applied parameters, e.g., the laser power (30 W), the scanning speed of the laser beam (from 50 mm/s up to 300 mm/s) and the minimum interline distance between the grooves (400 µm), did not affect the formation of adjacent grooves. This observation was principal during the discussion of the relations between the laser’s working parameters—the geometry of the grooves and the strength of the polyamide/aluminum joints. 

The experiment showed that decreasing the distance between groove lines (from 800 µm to 400 µm) had a marginal effect on the shear strength of the joint, but did have a small impact on stress during breaking.

Using just one pass of a low-power laser beam had a huge impact on the morphology of the engraved surface, and it was proven that the effect was connected with the scanning speed. Low speeds produced high and necking recasting with deep but blocked grooves, while fast speeds produced shallow grooves with low recasting, but no obstacles. The lap shear tests indicated that strong connections came from deep, unobstructed grooves with moderate recasting, which implied a middle ground in terms of the scanning speed parameter values.

Two-sided joints were found to be highly dependent on other parameters, mainly scanning speed. With the correct parameters (mainly appropriate scanning speed), a two-sided joint can outperform a one-sided joint characterized by the same surface area. Otherwise, the two-sided joint will provide a small increase in joint strength at the cost of a doubled surface area, as compared to a simple one-sided joint.

One of the major advantages of two-sided joints comes from their almost doubly high displacement at the breaking point. In other words, two-sided connections of metal parts overmolded with polyamide 6 GF 30 require a maintained load and more time to reach the breaking point. 

## Figures and Tables

**Table 1:** Laser-engraving parameters.

Parameter	Value				Unit
Wavelength	1064				nm
Frequency	30				kHz
Beam intensity	30				W
Pulse width	160				nm
Beam quality	M2 < 1.5				
Divergence angle	0.5				mrad
Spot size	<10				μm
Distance between grooves	0.4; 0.8				mm
Scanning speed	50	100	200	300	mm/s
Energy deposition	3.0	1.5	0.8	0.5	mJ/cm^2^
Fluence	356.5				J/cm^2^

**Table 2:** Sample variants defined by differences in laser working parameters.

Distance between Grooves (mm)	Scanning Speed (mm/s)
0.4	50
	100
	200
	300
0.8	50
	100

**Table 3:** Injection overmolding parameters.

**Injection**			**Holding**		
Injection speed (maximal)	130	mm/s	Holding pressure	60	bar
Injection speed (real)	90	mm/s	Holding pressure time Z2	4	s
Injection pressure	100	bar	Holding pressure time Z4	30	s
Intrusion time	0	s	Cooling time	30.4	s
**Plasticizing**			**Temperature set up**		
Screw rotation speed	75	rpm	Nozzle (first zone)	255	°C
Screw back(feed) position	57	mm	Barrel (second zone)	250	°C
Plastification pressure	1.5	bar	Barrel (third zone)	245	°C
Plasticizing time	25	s	Barrel (fourth zone)	240	°C
Screw diameter	22	mm	Mold	80	°C

**Table 5:** Results of lap shear tests.

Distance between Grooves	Laser Scanning Speed	Joint Type ^1^	MaximumShear Force	Shear Strength	Force at Break	Shear Stress at Break
(mm)	(mm/s)	I/II	(N)	(MPa)	(N)	(MPa)
0.8	50	I	744 ± 67.7	1.260 ± 0.073	541 ± 87.7	0.920 ± 0.152
		II	1180 ± 33.5	0.999 ± 0.288	743 ± 79.2	0.630 ± 0.048
0.8	100	I	695 ± 111	1.150 ± 0,.164	557 ± 107	0.924 ± 0.155
		II	1210 ± 155	1.070 ± 0.090	651 ± 148	0.575 ± 0.126
0.4	50	I	883 ± 216	1.780 ± 0.471	746 ± 280	1.490 ± 0.591
		II	1230 ± 47.5	1.060 ± 0.081	1070 ± 64	0.928 ± 0.087
0.4	100	I	745 ± 87.3	1.310 ± 0.148	658 ± 86.1	1.160 ± 0.167
		II	1330 ± 41.8	1.130 ± 0.091	910 ± 465	0.779 ± 0.406
0.4	200	I	622 ± 336	1.080 ± 0.571	522 ± 291	0.914 ± 0.502
		II	1410 ± 77.4	1.200 ± 0.060	1170 ± 316	1.010 ± 0.284
0.4	300	I	617 ± 200	1.100 ± 0.377	539 ± 183	0.980 ± 0.395
		II	1280 ± 39.7	1.100 ± 0.093	1190 ± 45	1.020 ± 0.088

^1^ Refers to one-sided joints (I) and two-sided joints (II).
